# Death of Dr. Thomas Mackie Smith

**Published:** 1852-02

**Authors:** 


THE MEDICAL EXAMINER.

PHILADELPHIA, FEBRUARY, 1852.

### OBITUARY.

Died, on the 21st ult., at his late residence on the Brandywine, Delaware, THOMAS MACKIE SMITH, M. D., aged 42 years, the brother and preceptor of one of the Editors of this Journal.

Few men live who possessed more of the confidence and affection of their fellow citizens, and better deserved these, than the lamented subject of this notice. In the midst of a scattered population, he fulfilled the duties of his arduous profession, with untiring assiduity, and unswerving faithfulness; and by many years of devoted service and the kindest attentions, secured not only high admiration for his medical skill, but the warmest love and gratitude from all who received the benefit of his labors, or were made acquainted with his uniform self-denial and perseverance. A whole community, among whom he has gone in and out, relieving the sick, supplying the wants of the destitute, comforting the afflicted, and showing kindness to all, now rise up and call him *blessed*, and thus show how well he deserved that title, which belongs to so many of his profession—"the beloved physician."

